# Retrospective review of medication allergy labeling among patients admitted to a tertiary hospital in Qatar

**DOI:** 10.5339/qmj.2023.sqac.18

**Published:** 2023-11-26

**Authors:** Rana M. Al-Adawi, Reem Elajez, Dana Bakdach, Dina Elgaily, Ahmed Karawia, Asmaa Mohamed

**Affiliations:** ^1^Pharmacy Department, Hamad General Hospital, Hamad Medical Corporation, Doha, Qatar Email: RAhmed4@hamad.qa; ^2^Pharmacy Department, Rumailah Hospital, Hamad Medical Corporation, Doha, Qatar

## Abstract

**Background:** Timely access to accurate, up-todate drug allergy information is critical to avoid potentially life-threatening adverse drug reactions (ADRs). However, the completeness and accuracy of allergy documentation remain a challenge. Inappropriate allergy documentation usually necessitates alternative treatments, increases costs, and may negatively impact patients’ outcomes.

**Objectives:** Review medication allergy labeling documentation, identify the most reported medication class, and describe allergic reactions based on the reported severity.

**Methods:** A retrospective cross-sectional audit including all medication allergy labeling documentation for patients admitted to Hamad General Hospital (HGH) from January-December 2022 was conducted. A list of patients with medication allergies was generated from the pharmacy system, which included patients’ demographics, medication names, documented allergy severity, and any other comments. The list was reviewed, and medications were categorized into different classes.

**Results:** 2856 allergy documentation for 2431 unique patients were identified and included in the analyses. The mean age of included patients was 43 years old, with 73.2% (1780) being females. Among the reported allergic reactions, 11.8% (336) were documented as severe allergic reactions, 51.1% (1457) were moderate, and 37.1% (1060) were mild. Antibiotics were the most common documented allergens, representing 42.1% of all reported allergies, followed by non-steroidal anti-inflammatory drugs (20.7%, n=591), and paracetamol (5.3%, n=151). Of all the reported allergies, only 6 (0.21%) cases had documented confirmatory allergy tests done. Further analysis of the reported allergies revealed that 1.2% (34) of the allergies had documentation to counteract the allergy labeling through either revised patient history or re-challenging. Despite such, allergy labeling was kept in the medical profile without proper de-labeling.

**Conclusion:** Allergy labeling documentation is a key to safe medication prescribing. However, standardized allergy documentation should be implemented to include a brief description and onset of the symptoms. Additionally, a safe de-labeling pathway should be adopted. Most of the allergy documentation was based on patients’ or family/parents’ reports, while actual allergies observed by a healthcare provider were limited.

